# Does Classifier Fusion Improve the Overall Performance? Numerical Analysis of Data and Fusion Method Characteristics Influencing Classifier Fusion Performance

**DOI:** 10.3390/e21090866

**Published:** 2019-09-05

**Authors:** Sandra Rothe, Bastian Kudszus, Dirk Söffker

**Affiliations:** Chair of Dynamics and Control, Faculty of Engineering, University of Duisburg-Essen, 47057 Duisburg, Germany; bastian.kudszus@stud.uni-due.de (B.K.); soeffker@uni-due.de (D.S.)

**Keywords:** information fusion, data properties, fusion method characteristics, performance influencing factors

## Abstract

The reliability of complex or safety critical systems is of increasing importance in several application fields. In many cases, decisions evaluating situations or conditions are made. To ensure the high accuracy of these decisions, the assignments from different classifiers can be fused to one final decision to improve the decision performance in terms of given measures like accuracy or false alarm rate. Recent research results show that fusion methods not always outperform individual classifiers trained and optimized for a specific situation. Nevertheless fusion helps to ensure reliability and redundancy by combining the advantages of individual classifiers, even if some classifiers are not performing well for specific situations. Especially in unexpected (untrained) situations, fusion of more than one classifier allows to get a suitable decision, because of different behavior of classifiers in this case. Nevertheless, there are several examples, where fusion not always improves the overall accuracy of a decision. In this contribution fusion options are discussed to overcome the problem to overcome the aforementioned problem and to define influencing factors on overall fusion accuracy. As a results requirements for good or guaranteed or possibly increased fusion performance and also suggestions denoting those options not leading to any kind of improvement are given. For illustrating the effects a practical example based on three characteristics of fusion methods (type of classifier output, use of these outputs and necessity of training) and four data properties (number of classes, number of samples, entropy of classes and entropy of attributes) are considered and analyzed with 15 different benchmark data sets, which are classified with eight classification methods. The classification results are fused using seven fusion methods. From the discussion of the results it can be concluded, which fusion method performs best/worst for all data sets as well as which fusion method characteristic or data property has more or less positive/negative influence on the fusion performance in comparison to the best base classifier.Using this information, suitable fusion methods can be selected or data sets can be adapted to improve the reliability of decisions made in complex or safety critical systems.

## 1. Introduction

The implementation of decision support systems within complex and safety critical applications strongly relies on the dependable accuracy of decisions. Considering the application of methods for data classification, the assessment of situations or conditions should be improved.

According to Reference [1], only two principal ways to achieve an increase in classification performance exist. At a first sight, it seems to be possible to further increase the capabilities of an already given classification algorithm, e.g., by tuning hyperparameter for specific applications. This is usually not feasible due to the individual limitations of each classification method. Therefore, the second way to improve the performance is the method of classifier fusion. Fusion solely relies on the subsequent combination of decisions derived by different so called base classifiers. The main advantage of this method is the exploitation of different classifier specific strengths in terms of their specific suitability for different forms of classification problems [2]. A major drawback of decision fusion is the possibility of impeding the overall system performance by combination of several single classifiers.

Regarding the vision of Internet of Things (IoT), using small internet-connected devices for data generation and analysis of information, fusion can lead to more reliable information. Data fusion first reduces size and dimension of data, optimizes the amount of data traffic and extract useful information from raw data [3], information fusion can lead to an enhancement of information completeness and quality [4]. Applying unreliable data sources can lead to unreliable IoT applications. As stated in Reference [4], IoT trust models are crucial for information fusion as well as for the success of IoT. In Reference [3] the importance of data fusion in different application fields for IoT is already stated. In this contribution, the requirements for reliable information fusion are discussed based on data and fusion method characteristics.

**Figure 1:**
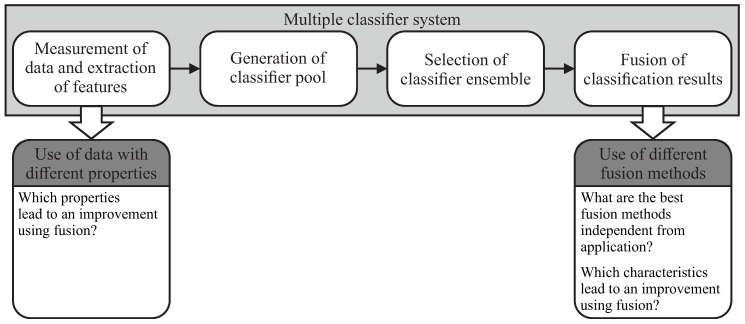
Scheme of a multiple classifier system and related research questions answered in this contribution.

Considering classifier fusion is part of a multiple classifier system, where the data measurement, feature extraction, generation of classifier pool and the Ensemble selection are done prior to the fusion itself (see Figure 1).

The choice of suitable classifiers for combination is one of the major problems within the field of classifier fusion. The achievable performance of fusion methods mainly relies on the selection of the most diverse and accurate single base classifiers [2]. According to Reference [5], no best classifier for all problems exist and the individual performance of classification methods itself also depends on several characteristics inherent to the classified set of data. In this contribution the possible relationships between different data and fusion method characteristics, with respect to the achievable performance of fusion methods are evaluated. The related questions to the individual parts of the multiple classifier system are shown in Figure 1.

Regardless whether suitable ensembles of classifiers have been chosen by application of static or dynamic selection methods, the parallel implementation of individual ensemble members requires further processing of results [6]. For this purpose, various methods fusing individually obtained decisions have been proposed in numerous works, for example, the Dempster-Shafer Combination [7], the Behavior Knowledge Space [8] or the Highest Rank [2], which tend to improve classification performance while forming a conclusive classification result.

Considering fusion methods, different characteristics can be selected for analysis. First the type of outputs generated by the individual classifiers forming an ensemble is an important property to be considered [9]. A further attribute distinguishing the fusion algorithms is the use of classifier outputs class-conscious or class-inherent [10]. The necessity of prior training of fusion parameters devides the fusion methods into trainable and not trainable methods [11].

In accordance with Reference [12], two of the defined different main categories of data inherent characteristics, referred to as simple and information theoretic measures, are considered in this contribution.

The following section, contains a brief overview of different fusion method characteristics as well as data properties within each of the aforementioned categories. The section concludes with a detailed theoretical description of the applied measures. In Section 3 the selected fusion algorithms, classifiers and data sets as well as the experimental procedure are explained. In Section 4 the results of the numerical calculations based on benchmark data with respect to the different considered characteristics are shown and discussed. Summary and conclusion are given in Section 5.

## 2. Considered Properties

### 2.1. Attributes and Requirements of Fusion Methods

Prior to elaborating different algorithms for decision combination, the attributes and requirements with respect to different methods of classifier fusion should be discussed. This section therefore is further subdivided into the description of different output levels, the different use of these outputs and the necessity of training of the inherent parameters of fusion methods [11].

#### 2.1.1. Type of Classifier Output Levels

Considering the utilized type of classifier output levels as an attribute of the considered fusion method, different definitions exist. According to Reference [13] the output levels can be divided into possibilistic, probabilistic and crisp labels. Using this notation, the possibilistic labels can be interpreted as possibility of class membership [14], the probabilistic labels as posterior probabilities [15] and the crisp labels denote an assignment of classifier for a single class. However, as the final output obtained by a specific classifier is not limited to a single class label a more practical categorization is proposed in Reference [16], which divides the different levels of classifier outputs into abstract, rank and measurement level. The abstract level is equivalent to the crisp labels but both possibilistic and probabilistic labels belong to measurement level. The rank level represents an ordered subset including the most plausible classes. Considering the amount of information inherent to each of the depicted categories, the least amount of information is provided on the abstract level, given the fact that only a single class label is generated without declaration of certainty or the information of alternative class labels [11]. In contrast, information based on the measurement level provide the highest amount of useful data, due to direct propagation of possibilistic or probabilistic labels as final classifier output [1]. However, caused by different mathematical backgrounds of classifiers, the application of information on measurement level often requires further normalization of results to ensure reasonable combination [2]. Transformation of an output with high level information, such as the measurement level, into an output of lower information density is always possible [1]. This can be justified by the fact that for the generation of an ordered set of possible class labels (i.e., rank level) as well as for the assignment of a single class label (i.e., abstract level) the amount of higher level information is merely reduced [16]. The transformation of output levels in direction of a higher information density is only possible, if additional data resulting from training of the individual classifier are available [1]. The similarity of all definitions for output labels is the clear distinction between abstract level (related to crisp labels) and soft level (related to rank and measurement level as well as possibilistic and probabilistic labels). In this contribution (in accordance with Reference [10]) the classifier output levels are distinguished into single class label outputs (abstract) and soft outputs (soft).

#### 2.1.2. Use of Classifier Outputs

The base classifiers assign either a support value for each class (rank, possibilistic or probabilistic) or a single class (support value equal to 1 for the supported class, all other values are zero) as output. Depending on how the fusion method is using the information of classifier outputs for final decision, the fusion methods are either denoted as class-conscious or class-indifferent [10]. The class-conscious fusion methods only use the support values of the considered class, neglecting the support values of the other classes. The class-indifferent methods include all support values into the decision process. According to Reference [10] class-conscious methods consider the class context but neglect some information given by the classifiers, whereas the class-indifferent methods use all information but ignore the context.

#### 2.1.3. Training of Fusion Method Parameters

According to Reference [17] fusion methods can be divided into trainable and fixed methods. The difference is the necessity of an additional training prior to the fusion process to set method-specific parameters. These different parameters could be weights associated with specific base classifiers, as done during application of Logistic Regression [2]. Another example is the training of conditional class probabilities computed in case of applying Bayes Belief Integration [16]. Approaches without any training of parameters are for example the Majority Voting or the Borda Count method to be used directly after classification. Considering fusion methods using training of parameters, an additional amount of data samples is required. As stated in References [17,18], the number of training data samples as well as the use of the same data for the training of base classifiers and fusion methods is significant in relation to the fusion results.

### 2.2. Data Characteristics

In this contribution the analysis of relationships between different data properties with respect to the usability of fusion methods is discussed. Given the fact that the achievable performance of the different base classifiers [12,19], and consequently the performance of the applied fusion methods, strongly depend on the characteristics inherent to the employed sets of data, the relevant characteristics of data sets should be discussed [12]. Two different main categories of data inherent characteristics are considered, referred to as simple and information theoretic measures.

#### 2.2.1. Simple Measures

The group of simple measures describes the basic characteristics of a single set of data. Simple measures can be combined in terms of proportions or products, and so forth, to generate additional information required for specific investigations [12]. As one of the simple measures describing the dimension of the underlying classification problem, the number of classes $n_{C}$ defines the number of different groups of instances within the considered set of data. A higher number of classes results in increasing complexity of knowledge representation. The number of classes characterizes the classifier and fusion performance. The number of samples or instances $n_{S}$ comprised by a single set of data increases on the one hand for a high number of samples the computational time during training of base classifiers but also enables a more detailed generation of knowledge. Thus larger data sets should theoretically tend to improve the overall performance of classification and fusion algorithms compared to the application of smaller sets of data.

#### 2.2.2. Information Theoretic Measures

The information theoretic measures are commonly related to the calculation of entropy denoting the mean information content. The entropy of two different relevant properties, the entropy of classes and entropy of attributes, are considered. The entropy of classes indicates the grade of evenness of the underlying class distribution. According to Reference [12], the entropy is calculated using

(1) $$H\left(C\right)=-\sum_{i}p_{i}\log_{2}p_{i},$$

where $\log_{2}$ defines the logarithm to basis two and $p_{i}$ represents the probability mass function for the class *i* (number of samples according to class *i* over total number of samples). For a random variable with equal probability for each of the possible values, the entropy reaches a maximum. A higher entropy of classes denotes that the number of samples according to each class is more even distributed. As the entropy represents the amount of attribute inherent information, for the entropy based on $\log_{2}$ the assigned unit is Bit. Considering the entropy of attributes, the formula is the same as for entropy of classes, only considering the class distribution according to one attribute *X*. For every attribute the entropy can be calculated. If one attribute only contains samples with the same class, the entropy is 0. The more even the classes are distributed within one attribute, the entropy increases. An attribute with zero entropy however, contains no information for class discrimination, due to the non existing variation between different instances of data [12]. To obtain one value for the data set, the mean value of the entropies calculated for each attribute is used.

## 3. Application Using Benchmark Data

To evaluate the influences of the above mentioned characteristics on the fusion performance, various experiments are conducted. Experiments instead of analytical calculations are used, because the final fusion result can not be calculated without specific assignments from the classifiers. These classifier assignments also depend on considered data sets [5], so that several assumptions and dependencies have to be considered. The experimental evaluation offers the advantage that these assumptions and dependencies are fixed for the given benchmark data sets. For example-related generalization training and test data sets are divided by nested cross-validation. By means of the experiments, conclusions and suggestions to the suitable fusion methods can be given.

The specification of considered fusion methods and base classifiers, as well as the definition of the within this study applied data sets and their fundamental properties is introduced first. The conducted experimental procedure as well as the intended purpose of several experimental design decisions will be detailed.

### 3.1. Fusion Algorithms

For a consistent distribution of fusion methods to all attributes, seven different fusion methods are selected. In Table 1, the methods Majority Voting (MV), Highest Rank (HR), Borda Count (BC), Bayes Belief Integration (BBI), Behavior Knowledge Space, Logistic Regression (LR) and Fuzzy Integral (FI) are shown with their specific characteristics according to the utilized type of classifier output, the necessity of training parameters and whether they are class-conscious (bold) or class-indifferent (not bold).

Majority Voting is based on the simple majority rule, where a selection or decision is made based on the number of votes for each alternative solution.

The Highest Rank method uses the best ranking of considered class as final ranking for fused results. Given the case that different class labels would receive an identical position in the final ranking, the conflicts are broken by random.

The fusion method Borda Count is an extension of MV, using rankings instead of specific class labels. Therefor, the so-called borda count is calculated for each class using the total number of classes ranked below the considered class and add this number for all classifiers. Using the resulting values in descending order, the final ranking can be set up according to the rank of the border count of the individual classes. Potential ties arising during the development of the final ranking are arbitrarily broken [20].

The Bayes Belief Integration also known as Bayesian Combination Rule or Bayesian Belief Method is a well known and commonly used fusion technique based on conditional probability. Based on the probability matrix of each classifier, a combined belief value for each class is determined for each sample.

The Behavior Knowledge Space method [8], uses the specific combination of classifier labels from a training data set to denote a most probable class for an unknown sample generating a new combination of classifier labels.

During Logistic Regression, the probability of a true class for a specific score vector is used to calculate the so-called empirical logits. Using the logistic response function and the formula for logits, model parameters can be estimated using methods related to linear regression. According to Reference [2], methods based on maximum likelihood or weighted least-squares can be used.

The application of Fuzzy Integrals within the field of classifier fusion, is often interpreted as searching for the maximum agreement between the individual classifiers decisions and a specific generated fuzzy measure for each class [21]. Finding these measures is the key problem which is solved with training data. Using the Sugeno Fuzzy Integral method (solely considered within this work), fuzzy densities can be calculated and the maximum degree of support denotes the fuzzy integral and thereby the final class.

### 3.2. Classification Methods

To generate classification results for fusion, the open source software package WEKA [22] is used to apply the classification methods. The different hyperparameters of each classifier are not considered, so the default-parametrization is used. The optimization of classifier inherent parameters is a highly problem specific task [23]. Different data characteristics usually require different parameter adjustments, so the application of optimized models on differing data sets would lead to highly biased results, when not specifically optimized for each of the given problems itself [24]. An optimization process requires additional amount of data, which is often unfeasible due to the already limited number of samples available. Therefore, to bypass the problem of limited data and as done in several other contributions ([5,25]), the applied classifiers are implemented with the default parameter setting applying the WEKA machine learning toolbox version 3.8.2 [22]. The classification methods to be discussed are C4.5 Decision Tree, Multilayer Perceptron, Radial Basis Function Network, Naive Bayes, K-Nearest Neighbors, Support Vector Machine, Expectation Maximization and Simple K-Means. To enable the application of unsupervised clustering methods with respect to the problem of classification, the WEKA ClassificationViaClustering procedure [22] is implemented, which generates a mapping between the clusters derived from training data and their corresponding class labels in a supervised manner [26].

### 3.3. Data Sets

A large amount of potentially useful measures are known to describe data characteristics. To cover a preferably wide range of characteristics, the conducted experiments are based upon 15 different problems taken from the UEA & UCR Time Series Classification Repository [24]. Selected data sets and corresponding characteristics are listed in Table 2. All selected data sets are characterized by nominal class labels and continuous valued numerical attributes without any missing values present. Each of the data sets has been reviewed prior to application in terms of standardization, since incorrect or nonexistent standardization can affect the results during classification [24]. Each of the selected data sets originally contains standardized samples of zero mean and unit standard deviation.

### 3.4. Experimental Procedure

In this contribution dependencies between different data and fusion method characteristics with respect to the usability of fusion methods should be detected. Although several measures for assessing the performance of classification algorithms exist [23], the focus of the conducted experiments is given to the analysis with respect to the overall classification accuracy obtained by classifier fusion. This section details the applied procedure based on the aforementioned methods and data sets.

#### 3.4.1. Nested Cross Validation

According to k-fold cross validation, data sets are divided into k folds. Random partitioning of data into *k* disjoint folds is done [25]. The partitioning conducted within each of the outer loops of cross validation is the same for every classifier and fusion method (due to implementing seeded random partitioning in WEKA) [26]. This way, by generating the exact same folds of data for every classifier and fusion method, all of of the conducted experiments are completely reproducible when based upon the attached basic partitions of data. To prevent additional bias caused by a dissimilar deployment of classes between training and test data, each of the derived partitions possesses the same class distribution as the corresponding original set of samples by implementing stratification of partitions [26,27]. The suggested number of folds using cross validation according to investigations in References [28,29], strongly depends on the stability of applied induction algorithms. For larger values of *k* (10–20), in Reference [28] a reduced variance by simultaneous increasing bias of estimates is noted, while for smaller values (k=2) the variance increases significantly. In a similar way, the recommendations proposed in Reference [29] ranges between two to ten folds. Here the applied number of folds is consequently defined as k=5 for both inner and outer loop of cross validation, providing a trade-off between bias and variance while restricting computational effort. The considered data set is divided into 5 folds, four of these folds are used to train, one fold is used to test the classifiers. Using the test fold, also the fusion methods are applied to compare the fusion performance with the individual classifier performance. This training/test procedure is repeated five times. According to Reference [30], the resulting mean value (here of gain of accuracy) is used to compare the performances.

As explained, some of the selected fusion methods need also an additional training prior to the fusion process. Following the contributions of References [30,31,32] generating unbiased performance estimates, every aspect of parameter tuning or selection should be included within the procedure of cross validation itself, so a nested approach of cross validation is considered. From all calculated values, the mean value is set as the final parameter used in the fusion process.

#### 3.4.2. Performance Measure

To compute a measure for improvement obtained by fusion of all classifiers, for each set of data as well as fusion method, the accuracy of the best individual classifier is compared to the accuracy of the fused ensemble itself. The partitioning of every data set is exactly the same for all of the considered classifiers and fusion methods, the comparison of accuracy is conducted in accordance with the matched sample approach suggested by Reference [32].

Therefore, denoting *n* as the number of samples of a single data set *Z*, the applied partitioning of *Z* into *k* disjoint folds $F_{j}$ with j∈{1,…,k} leads to a reduced number of samples *m* within each fold with n=k·m [32]. Based on the number of correctly predicted samples in the current test fold denoted by $r_{best,j}$ and $r_{ens,j}$ for the best classifier and the considered fusion algorithm respectively, the gain of accuracy $\Delta ACC_{j}$ for each fold $F_{j}$ can be computed by

(2) $$\Delta Acc_{j}=\frac{r_{ens,j}-r_{best,j}}{m}.$$

Based upon the *k* different results obtained for each of the derived partitions $F_{j}$, the estimated enhancement of accuracy for a specific fusion algorithm with respect to dataset *Z* [32] is further evaluated by

(3) $$\Delta Acc=\sum_{j=1}^{k}\frac{\Delta Acc_{j}}{k}.$$

Finally the classification performance enhancement is consecutively plotted against the different characteristics to investigate the impact of the mentioned method-related properties of classifier fusion and data characteristics.

## 4. Experimental Results

To evaluate the performance improvement and the influences of different characteristics, the results are discussed in the following. First the overall performance will be shown. The results related to the characteristics of fusion methods and data sets are discussed subsequently.

### 4.1. Mean Performance of Fusion Methods

**Figure 2:**
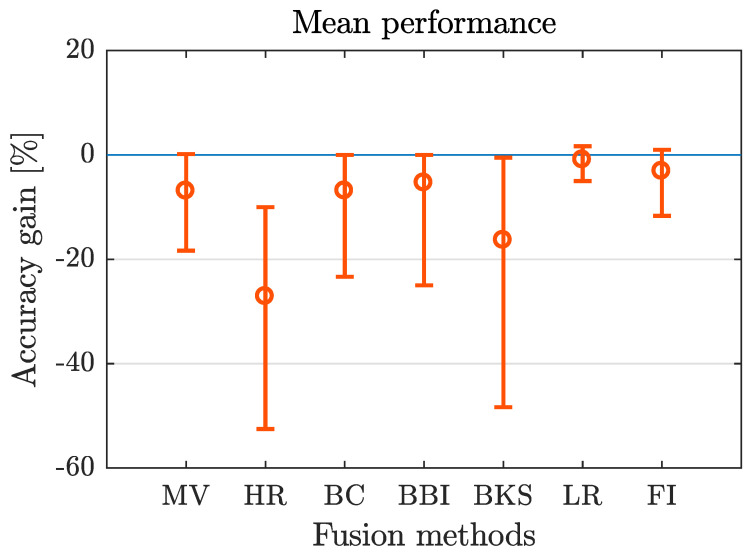
Mean, minimum and maximum accuracy gain over all data sets for each fusion algorithm applied.

To analyze the overall fusion performance, the accuracy gain calculated for each fusion method and data set is shown in Table 3. For all 105 combinations of data set and fusion method, only 13 times no deterioration compared to the best individual classifier occur. Considering the number of best and worst results, LR is the fusion method with the best performance (12 times best result out of 15 data sets). This is also confirmed calculating the mean percentage (−0.95%) over all considered data sets (Table 3 last row). The second best results are produced using FI (mean percentage =−3.14%). The mean percentage for the methods BBI, BC and MV are in a similar range (−5.37% to −6.94%). The fusion method HR shows the worst results for the considered data sets, for 10 out of the 15 data sets as well as for the mean percentage, HR produces the least accuracy gain. The BKS shows worst results for the remaining 5 data sets. In Figure 2 the mean percentage as well as minimum and maximum value of accuracy gain are plotted for different fusion methods.

The results show that also the range between minimum and maximum value is lower for those fusion methods with better mean value (LR and FI) and higher for those with worse results (BKS and HR). Although there are strong tendencies for specific fusion methods, the variety of results is very high, not only for the different fusion methods, also for different data sets. The dependency of the results to the different characteristics of fusion methods and data sets is analyzed in the following.

#### Lessons Learned

In most cases fusion led to a deterioration in accuracy compared to the best individual classifier. The LR fusion method leads to the best, the HR to the worst results regarding the considered data sets and fusion methods.

### 4.2. Performance Related to Fusion Method Characteristics

**Figure 3:**
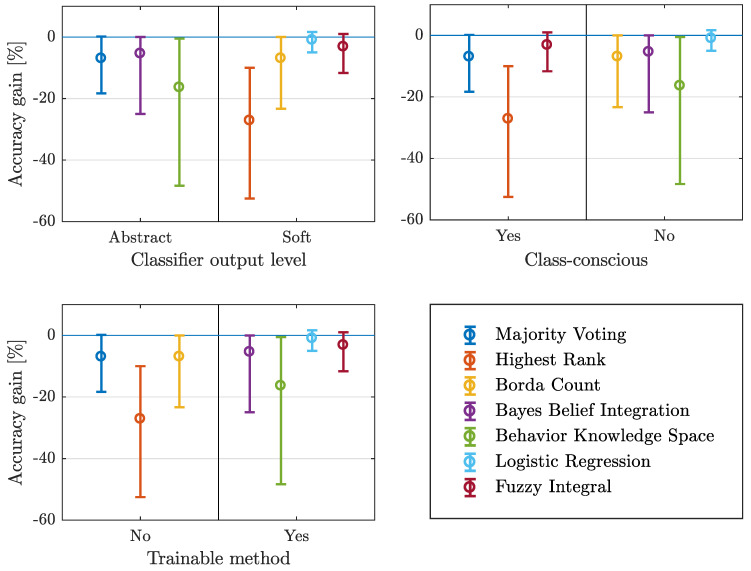
Mean, maximum and minimum accuracy gain over different characteristics of fusion methods.

The distinction between trainable and non trainable, class-conscious and class-indifferent, as well as abstract and soft level-supported methods, is illustrated in Figure 3.

Considering the type of classifier output, which is used in fusion methods, it cannot be stated that one type outperforms the other (see Figure 3 top left). Fusion methods with the best and worst results (LR and HR) both use soft classifier outputs. Further, the mean value of the individual mean values (from Table 3) is similar in both categories (abstract: −9.56%, soft: −9.53%). For the considered data sets and fusion methods, the type of classifier output has no significant influence although the information content is higher using the soft level.

Beside the mentioned type of classifier outputs, also the influence of different use of these outputs is considered. In Figure 3 top right the results are distinguished in class-conscious and class-indifferent fusion methods. The results show that the two best performances produced by LR and FI, as well as the two worst results produced by BKS and HR are in different categories, whereas the best performance is produced by a class-indifferent method, the worst by a class-conscious one. The mean of the individual mean values also show a difference (class-conscious: −12.41%, class-indifferent: −7.40%) and a tendency to class-indifferent methods.

Taking the necessity of an additional training into account, the best three performance values can be reached using trainable fusion methods. The mean of individual mean values show a smaller absolute value for trainable methods (not trainable: −13.67%, trainable: −6.47%). This result shows that additional training, providing additional information used in the fusion process, leads to better results.

#### Lessons Learned

The level of classifier output does not have significant influence on the fusion performance. Class-indifferent and trainable methods perform slightly better than class-conscious and non trainable methods.

### 4.3. Performance Related to Data Characteristics

The following paragraphs focus the impact of data inherent characteristics. Therefore the mean, minimum and maximum values of accuracy gain are plotted for each fusion method and data set separately over the considered data properties.

#### 4.3.1. Number of Classes

**Figure 4:**
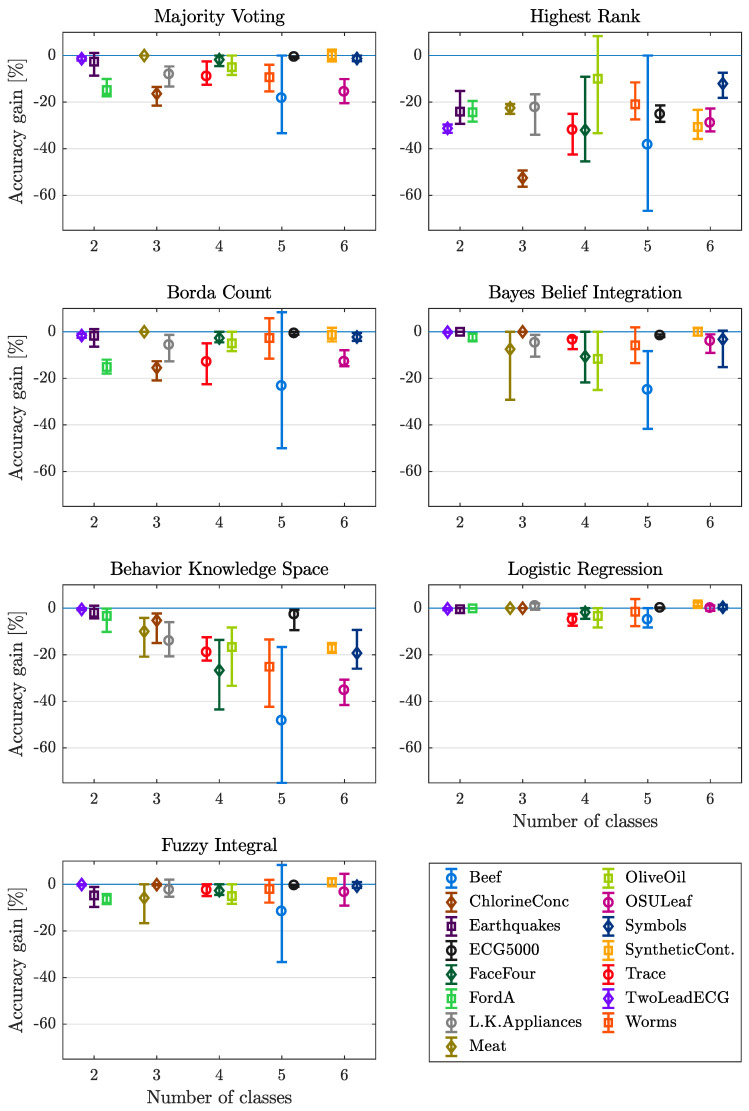
Mean, minimum and maximum gain in accuracy over number of classes, with respect to each dataset and fusion method considered.

The first data characteristic analyzed is the number of classes. In Figure 4 each plot contains the accuracy gain for the 15 data sets depending on their number of classes of one fusion method. The results show that the number of classes has not an influence on every fusion method. The methods MV, HR, BC and FI do not show a significant change in mean value, only the range between maximum and minimum value increases and reaches its maximum at 5 classes considering the methods HR and BC. Utilizing the fusion methods BBI, BKS and LR, a decrease of accuracy gain as well as an increase of the range can be observed. For the LR method, the occurrence of this tendency is not as significant as for the BBI and BKS fusion methods. Both methods (Bayes Belief Integration and Behavior Knowledge Space) rely on the previous training of method inherent parameters. In the case of BBI, the probability matrix which has to be computed prior to classification, grows quadratic with the number of classes. Thus an increasing number of classes requires a higher number of training samples. In a similar way the application of Behavior Knowledge Space relies on the previous computation of probabilities for each of the possible combinations of labels generated by the different base classifiers. Hence the knowledge space also grows exponentially with the number of possible classes, which in the case of restricted data for training also impedes derivation of proper results.

#### Lessons Learned

An increasing number of classes (up to 5 classes) leads to decreasing performance of most of the considered fusion methods. For a small number of classes, BBI, BKS, LR and FI are suitable but for a higher number of classes, only LR and FI are recommended.

#### 4.3.2. Number of Samples

**Figure 5:**
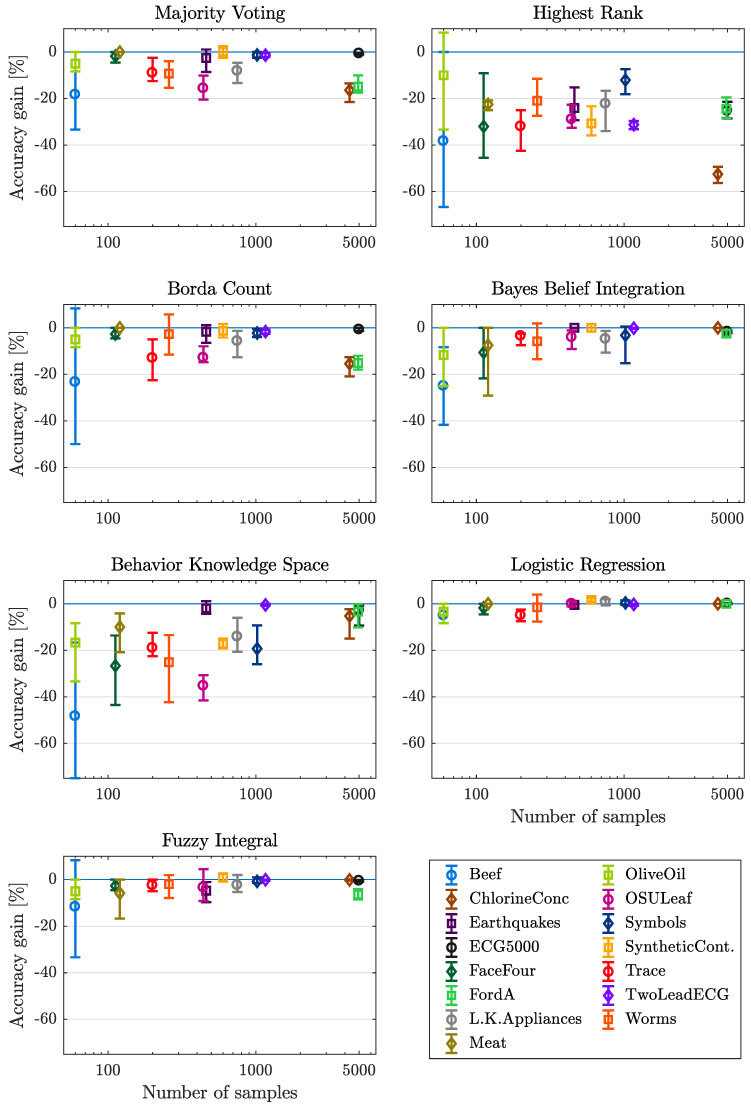
Mean, minimum and maximum gain in accuracy over number of samples, with respect to each dataset and fusion method considered.

In Figure 5 the accuracy gain is plotted over the number of samples provided by the individual data set. Here the number of samples of the applied data sets ranges from 60 samples (Beef and OliveOil) to 5000 samples (ECG5000). The x-axis is scaled logarithmic because of a slight majority of lower numbers. The mean values of accuracy gain reached with the fusion methods MV, HR, LR and FI show no clear tendency for increasing number of samples except of the data set ChlorineConc. (4307 samples) using HR method. Considering the methods BC, BBI and BKS the loss of accuracy decreases for increasing number of samples. Regarding the range between minimum and maximum accuracy gain, the range decreases for all methods except of LR for increasing number of samples. Some exceptions for some data set/fusion method combinations should be stated: The data sets FaceFour (112 samples) and Meat (120 samples) show a small range and also a higher mean value using the fusion methods MV and BC, also showing a small number of samples. All exceptions only appear for fusion methods, where no additional training of fusion parameters is necessary. The most drastic impact can be noticed for the methods of BBI as well as BKS, with an improvement of over 20% and 40% respectively.

#### Lessons Learned

All trainable methods show increasing performance for increasing number of samples. If a large amount of data is available, trainable methods should be preferred, if only a small number of samples can be used, LR or FI should be applied.

#### 4.3.3. Entropy of Classes

**Figure 6:**
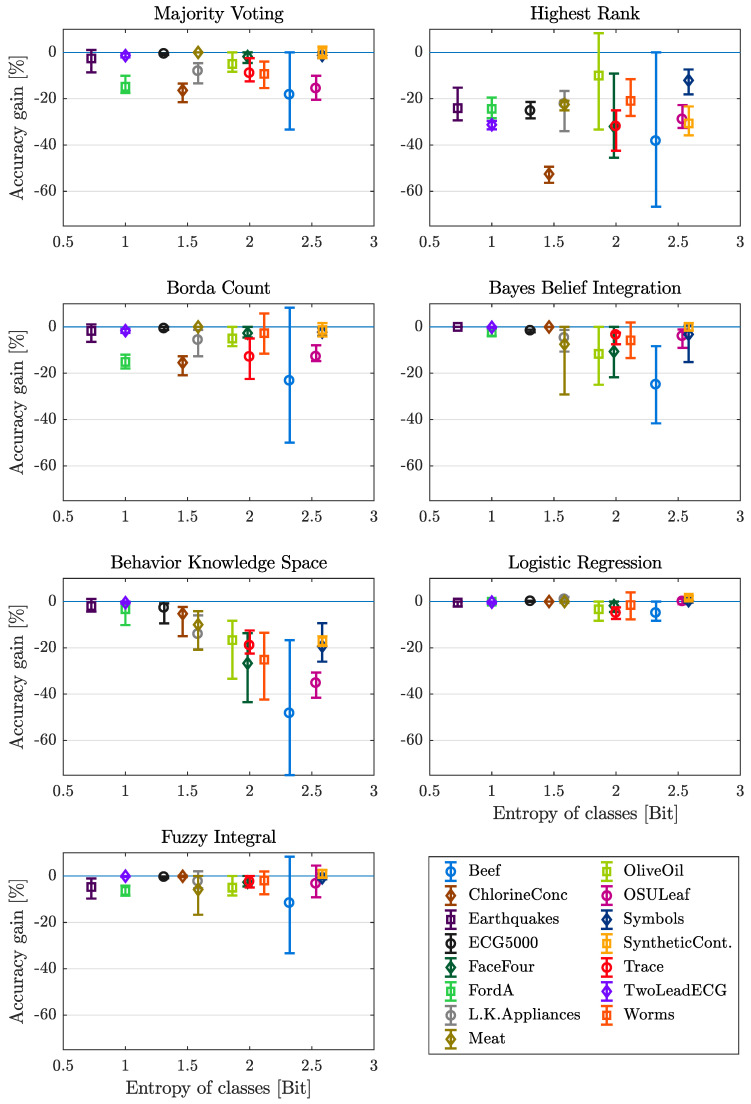
Mean, minimum and maximum gain in accuracy over entropy of classes, with respect to each dataset and fusion method considered.

To evaluate the dependency of fusion results on the evenness of class distribution, the accuracy gain is plotted against the entropy of classes for each data set and fusion method in Figure 6. The within this work classified sets of data comprise a range of entropy between 0.7245 to 2.5850 Bit for the Earthquakes and SyntheticControl dataset respectively. Regarding the methods BBI, BKS and LR the results show a more or less significant influence on the mean value of accuracy gain. With increasing entropy, the mean value decreases exept of data sets with entropy of more than 2.5 Bit (OSULeaf, Symbols and SyntheticControl). The most clearly observable tendency can be recognized for the method of BKS. The methods MV, HR, BC and FI do not show a tendency of mean value for changing entropy of classes. The range between minimum and maximum value increases with increasing entropy. This can be observed for all methods, the most drastic impact on maximum-minimum range can be noticed for the methods of BKS, BBI, BC and HR. The entropy of classes, as a measure of class distribution, reaches the highest value with respect to a specific number of possible classes, if all of the possible labels are represented by the same amount of samples within the considered set of data. A higher value of entropy corresponds to a higher probability for the samples of a specific class, to take part in the set applied for inducing the models for classification. The fact that skewed sets of data in many cases tend to cause overly optimistic results in terms of the resulting accuracy, may explain the observed behavior for certain fusion methods.

#### Lessons Learned

An increasing entropy of classes leads to a decreased performance for most fusion methods, although the information content is higher and the classes are more even distributed for high entropy of classes. For a high entropy of classes, only LR and FI show good performance, while only HR is not recommended to be implemented for data with small entropy of classes.

#### 4.3.4. Entropy of Attributes

**Figure 7:**
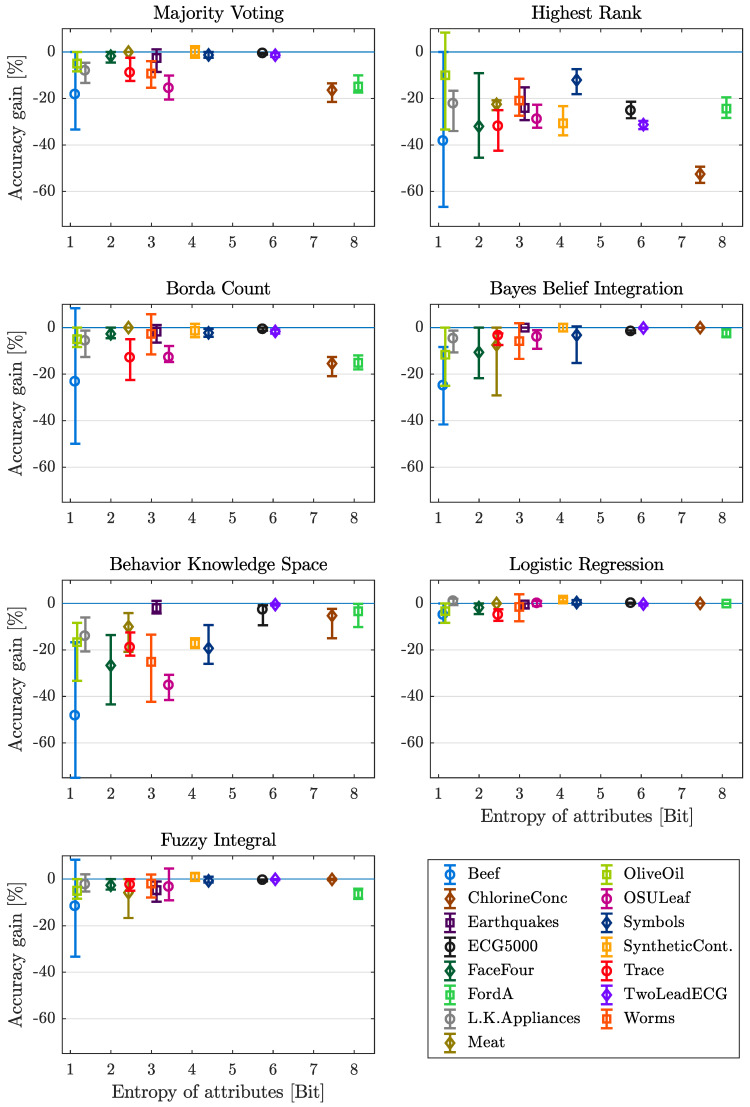
Mean, minimum and maximum gain in accuracy over mean entropy of attributes, with respect to each dataset and fusion method considered.

Not only the entropy of classes, also the entropy of attributes is considered in this contribution. As mentioned, for each attribute of one data set, one entropy value is calculated. To get one value specific for one data set, the mean value of the entropy of all attributes is calculated. The within this work selected sets of benchmark data, comprise a range of entropy from 1.1211 Bit to 8.0988 Bit for the Beef and FordA dataset respectively. The mean, maximum and minimum of accuracy gain in dependency of the entropy of attributes is shown in Figure 7 for each data set and fusion method. Considering the mean value of accuracy gain, a small increase in mean for increasing entropy of attributed can be observed using the methods MV, BC and BBI, whereas using the BKS fusion method, the influence is significant. The other methods (HR, LR and FI) show evenly distributed mean values for all entropies. The data sets ChlorineConc and FordA show the highest entropy of attributes but the mean of accuracy gain deceases significantly for one or both of these data sets when using the fusion methods MV, HR or BC (again only the not trainable methods). For all fusion methods the range between minimum and maximum decreases significantly for increasing entropy of attributes. The mean entropy of attributes is defined as the arithmetic mean over all single attributes entropy. Given the fact that an attribute with an corresponding low value of entropy comprises less change in magnitudes, these attributes tend to provide only a slight amount of additional information usable for the task of classification [12]. The in Figure 7 illustrated results support these quotations, since for lower values of mean entropy and therefore less attribute inherent information, the performance of the applied methods of decision fusion is clearly impaired.

#### Lessons Learned

An increasing performance can be observed for increasing entropy of attributes, because more information can be extracted using data sets with a high entropy of attributes. Considering data with a low entropy of attributes, only LR should be used as the fusion method. For data with higher entropy of attributes, LR and also BBI, BKS and FI can be recommended.

### 4.4. Are Fusion Methods Improving the Overall Performance? Conclusions from the Numerical Analysis

The question of whether fusion methods increase the overall performance and which characteristics are influencing the fused performance is considered. A summary of all lessons, which can be generated from the numerical analysis, are listed in Table 4.

The overall fusion performance illustrates the differences between the considered fusion methods. While the Logistic Regression outperforms the other fusion methods, the performance of the best individual base classifier can only be exceeded in 5 out of 105 cases. In 92 of 105 cases, using fusion leads to a deterioration of performance compared to the best individual classifier performance.

The type of classifier output (abstract or soft), as well as how these outputs are used (class-conscious or class-indifferent), have no significant influence on the fusion performance, whereas the trainable methods show slightly better performance than methods without the additional training prior to the fusion process.

Considering data characteristics, the results show that a higher number of classes leads to worse performance for some of the fusion methods. The methods LR and FI show the most constant performance for all numbers of classes, while BKS shows the highest sensitivity to increasing class number.

The more samples the data set have, the more information can be used for training the base classifiers as well as the parameters of fusion methods if necessary. This results in a better performance of the trainable methods for data sets with higher number of samples and can be concluded from results by the increasing mean value only for trainable methods (BBI, BKS, LR and FI).

Although a higher entropy of classes denotes more even distributed classes in the considered data set, a decrease in performance (mean and also range between maximum and minimum accuracy gain) can be observed for most of the fusion methods. Considering data sets with low entropy, all fusion methods except of HR (fusion method with worst overall performance) show similar and good performance. Increasing the entropy of classes, only using LR and FI are recommended to reach good performance.

Complementary to the entropy of classes, the higher entropy of attributes leads to the better performance of fusion methods. The higher the entropy, the more information can be extracted from the attributes, which can also be concluded from the results of all fusion methods.

Considering all data characteristics, MV, LR and FI show the least sensitivity to a change in these characteristics. While LR is performing best in the overall performance, the absolute influence of the changes is the least using LR. Hence the LR method is denoted as the least sensitive method. The fusion method BKS is most sensitive to changes of applied data characteristics.

## 5. Summary and Conclusions

The aim of this contribution conducted is to investigate relationships between fusion methods and data characteristics. The question to be answered is: Which fusion method improves in which case the overall performance? Therefore 15 different sets of benchmark data with different number of classes and samples as well as different entropy of classes and attributes were classified by eight base classifiers implemented using the WEKA machine learning toolbox. The generated classification results were fused by seven selected fusion algorithms. The fusion methods use different types of classifier outputs (abstract and soft) in different ways (class-conscious or class-indifferent). Some need additional training prior to fusion, some not. During experiment, nested 5-fold cross validation is used to distribute the data sets into training and test for classifiers and training and validation for fusion methods to obtain representative and (with the given restrictions) generalized results.

The main and most important result is that in most cases the use of fusion methods do not outperform the maximum individual classifier performance. However, the use of fusion has advantages like insensitivity to overfitting or redundancy. The results of this numerical analysis leads to the conclusion that the fusion performance strongly depends on the individual fusion method in combination with data characteristics. Some principal connections are carried out and given as recommendations.

In this contribution the accuracy as a measure for the overall performance is considered. In further considerations, also measures like recall, precision, f-score or false alarm rate can be considered to evaluate the effects to individual classes or assignments.

## Figures and Tables

**Table 1:** Different fusion methods ordered by characteristics (class-conscious methods are printed in bold) (referring to Reference [10]).

Output Level	Trainable Method	
	No	Yes
Abstract	**Majority Voting**	Bayes Belief Integration Behavior Knowledge Space
Soft	**Highest Rank** Borda Count	Logistic Regression **Fuzzy Integral**

**Table 2:** Benchmark data selected from the UEA & UCR Time Series Classification Repository [24].

Name of Dataset	Samples	Classes	Data Origin
Beef	60	5	Spectrograph
ChlorineConcentration	4307	3	Simulated
Earthquakes	461	2	Vibrations
ECG5000	5000	5	ECG measurement
FaceFour	112	4	Images
FordA	4921	2	Engine noise
LargeKitchenAppliances	750	3	Energy consumption
Meat	120	3	Spectrograph
OliveOil	60	4	Spectrograph
OSULeaf	442	6	Images
Symbols	1020	6	Images
SyntheticControl	600	6	Simulated
Trace	200	4	Simulated
TwoLeadECG	1162	2	ECG measurement
Worms	258	5	Motion

**Table 3:** Mean accuracy gain in percent for each fusion method and data set. The best as well as the worst result for each considered data set is printed in bold or is underlined respectively, not negative values are highlighted in green.

Data Set/Fusion Method	MV	HR	BC	BBI	BKS	LR	FI
Beef	−18.33	−38.33	−23.33	−25.00	−48.33	**−5.00**	−11.67
ChlorineConcentration	−16.39	−52.52	−15.46	−0.02	−5.27	**0.00**	−0.14
Earthquakes	−2.59	−24.07	−1.73	**0.00**	−1.95	−0.43	−4.76
ECG5000	−0.66	−25.30	−0.76	−1.68	−2.78	**0.08**	−0.52
FaceFour	**−1.78**	−32.02	−2.69	−10.63	−26.68	**−1.78**	−2.69
FordA	−14.83	−24.36	−15.14	−2.48	−3.35	**−0.06**	−6.38
LargeKitchenAppliances	−8.13	−22.27	−5.73	−4.80	−14.13	**0.93**	−2.40
Meat	**0.00**	−22.50	**0.00**	−7.50	−10.00	**0.00**	−5.83
OliveOil	−5.00	−10.00	−5.00	−11.67	−16.67	**−3.33**	−5.00
OSULeaf	−15.63	−28.95	−12.89	−4.08	−35.28	**0.00**	−3.42
Symbols	−1.27	−12.06	−2.25	−3.24	−19.31	**0.39**	−0.78
SyntheticControl	0.17	−30.67	−1.33	0.00	−17.17	**1.67**	1.00
Trace	−9.00	−32.00	−13.00	−3.50	−19.00	−5.00	**−2.50**
TwoLeadECG	−1.38	−31.24	−1.63	−0.17	−0.52	−0.26	**−0.09**
Worms	−9.28	−20.94	−2.71	−5.81	−25.13	**−1.52**	−1.95
Mean percentage	−6.94	−27.15	−6.91	−5.37	−16.37	**−0.95**	−3.14

**Table 4:** Lessons learned from the numerical analysis.

Characteristic	Lessons Learned
Overall performance	In most cases fusion leads to a deterioration in accuracy compared to the best individual classifier. The LR fusion method leads to the best, HR to worst results regarding the considered data sets and fusion methods.
Classifier output level	The level of classifier output does not have significant influence on the fusion performance.
Use of classifier output	Class-indifferent methods perform slightly better than class-conscious methods.
Necessity of training	Trainable methods perform slightly better than non trainable methods.
Number of classes	For most fusion methods an increasing number of classes (up to 5 classes) leads to decreasing performance. For a small number of classes, BBI, BKS, LR and FI are suitable but for a higher number of classes, only LR and FI are recommended.
Number of samples	All trainable methods show increasing performance for increasing number of samples. If a large amount of data is available, trainable methods should be preferred, if only a small number of samples can be used, LR or FI should be applied.
Entropy of classes	An increasing entropy of classes leads to a decreased performance for most fusion methods, although the information content is higher and the classes are more even distributed for high entropy of classes. For a small entropy of classes, only LR and FI show good performance, while only HR is not recommended to be implemented for data with higher entropy of classes.
Entropy of attributes	An increasing performance can be observed for increasing entropy of attributes, because more information can be extracted using data sets with a high entropy of attributes. Considering data with low entropy of attributes, only LR should be used as fusion method. For data with higher entropy of attributes, LR and also BBI, BKS and FI can be recommended.
Sensitivity to all data characteristics	Using the fusion method LR, the results are at least sensitive to the changes in the data characteristics, while BKS shows the most sensitivity.
